# Lecanemab approval in EU: what should we be ready for?– the EANM perspective

**DOI:** 10.1007/s00259-025-07066-9

**Published:** 2025-01-10

**Authors:** Igor Yakushev, Antoine Verger, Matthias Brendel, Diego Cecchin, Pablo Aguiar Fernandez, Francesco Fraioli, Timo Grimmer, Nelleke Tolboom, Tatjana Traub-Weidinger, Eric Guedj, Donatienne Van Weehaeghe

**Affiliations:** 1 Department of Nuclear Medicine, School of Medicine, TUM University Hospital, Technical University of Munich, Klinikum rechts der Isar Ismaninger Str. 22, 81675 Munich, Germany; 2 Department of Nuclear Medicine and Nancyclotep Imaging Platform, CHRU Nancy, Université de Lorraine, Nancy, France; 3 Department of Nuclear Medicine, LMU Hospital, LMU Munich, Munich, Germany; 4 Department of Medicine, Unit of Nuclear Medicine, University Hospital of Padova, Padova, Italy; 5 CIMUS, Universidade Santiago de Compostela & Nuclear Medicine Department, Univ. Hospital IDIS, Santiago de Compostela, Spain; 6 Institute of Nuclear Medicine, University College London, London, UK; 7 Department of Psychiatry and Psychotherapy, School of Medicine, TUM University Hospital, Technical University of Munich, Munich, Germany; 8 Department of Radiology and Nuclear Medicine, University Medical Centre Utrecht, Utrecht, The Netherlands; 9 Department of Diagnostic and Therapeutic Nuclear Medicine, Clinic Donaustadt, Vienna Health Care Group, Vienna, Austria; 10 Département de Médecine Nucléaire, Aix Marseille Univ, APHM, CNRS, Centrale Marseille, Institut Fresnel, Hôpital de La Timone, CERIMED, Marseille, France; 11 Department of Radiology and Nuclear Medicine, Ghent University Hospital, Ghent, Belgium

Lecanemab, a humanized IgG1 monoclonal antibody, binds to both insoluble and soluble amyloid-beta (Aβ) aggregates and, by doing so, reduces Aβ plaques in the brain in a cumulative dose- and time-dependent manner.

The U.S. Food and Drug Administration (FDA) approved this drug for treating Alzheimer’s disease (AD) in January 2023. The FDA approval, alongside with underlying clinical trials, has been commented by the EANM Neuroimaging Committee elsewhere [1]. Following the initially negative vote in July 2024, the Committee for Medicinal Products for Human Use (CHMP) of the European Medicines Agency (EMA) recommended granting a marketing authorization to Lecanemab for treating mild cognitive impairment (MCI) and mild dementia due to AD in November 2024 [2]. Importantly, this authorization is limited to patients with no or *one* copy of the apolipoprotein E ε4 (ApoE ε4) gene, the primary genetic risk factor for sporadic AD. These individuals have a lower incidence of amyloid-related imaging abnormalities (ARIA), a side effect of anti-amyloid antibodies [3], relative to ApoE ε4 homozygotes. As the therapy efficacy does not differ, the CHMP concluded that benefits of Lecanemab outweigh the risks in patients with one or no copy of the ApoE ε4. This population comprises 80 to 85% of patients with MCI and mild dementia due to AD.

Lecanemab has already been approved in the United States, Japan, China, South Korea, Hong Kong, Israel, Taiwan, the United Arab Emirates, and the Great Britain, and is being marketed in the United States, Japan, and China. Following the European Academy of Neurology [4], we welcome this positive vote of the CHMP. The CHMP’s opinion marks an intermediate phase in Lecanemab’s route towards patient accessibility. This recommentation will be forwarded to the European Commission, which will decide on an EU-wide marketing authorisation in January 2025. Once granted, pricing and reimbursement decisions will be made by each EU member state, considering the potential application and role of this medication within their national health systems.

Therapy with Lecanemab requires evidence of Aβ pathology that can be supplied with both amyloid PET and lumbar puncture (LP) for cerebro-spinal fluid (CSF) amyloid proteins. Whereas the former is more expensive, the latter is more invasive. The methods have a similar diagnostic accuracy [5], albeit amyloid PET provides greater changes in diagnosis and diagnostic confidence [6, 7]. Furthermore, amyloid PET is stronger associated with cognitive outcomes than CSF amyloid proteins [8, 9]. In our opinion, and as recommended elsewhere [10], the choice between LP and PET should be based on availability, level of experience and standardisation at a given center. Additionally, some patients may refuse LP in favour of PET. In this context it is notable that CSF and PET are equally treated as biomarkers of Aβ pathology by recent diagnostic guidelines on AD [11, 12]. Even in a scenario where LP is performed first [13], amyloid PET has a significant added value. This is in particular the case when CSF findings are close to a threshold, around 15–20% in specialized memory centers [14, 15], and in the case of a mismatch between the primary clinical diagnosis and CSF results [15].

Another advantage of amyloid PET, the marker of *aggregated* Aβ, is the ability to monitor response to anti-amyloid therapies. Fluid biomarkers, being sensitive to dynamic changes of amyloidopathy, are not recommended for monitoring amyloid load under disease-modifying therapies. Thus, amyloid PET rather than CSF Aβ proteins have been used as a secondary end point in clinical trials of anti-amyloid therapies so far [16]. As discussed previously [1], we foresee a relevant potential for repeated amyloid PET to guide therapy dosage and duration. In this context, adaptation of the centiloid scale to harmonize the quantification of amyloid PET images with the different radiotracers is essential [17]. In fact, the centiloid scale has been used to select and *monitor* patients in a prospective phase 3 trial of Donanemab, another promising anti-amyloid antibody [18], which is currently under review by the EMA. Specifically, Aβ load reduction was re-evaluated using PET at 24 and 52 weeks. Patients receiving Donanemab were transitioned to a placebo if PET quantification at these intervals met predetermined stopping criteria, which was achieved by 52% of patients in the treatment group [18]. Considering the significant patient burden and costs of Aβ-targeting monoclonal antibodies, a limited duration of therapy could substantially improve cost-effectiveness [19]. While the standard pipeline for calculation of the centiloid values seems to be robust [20], caution needs to be made regarding the potential influence of reconstruction PET parameters on the centiloid value [21].

Recent advances in blood-based biomarkers such as phosphorylated tau 217 hold promise for broadening access to biomarker-driven diagnosis [22]. Blood tests are minimally invasive and cost-effective, though challenges remain with standardization, sensitivity, and specificity [23]. At present, blood-based biomarkers, once approved, may serve as screening tools, but more evidence is needed to qualify for selecting patients for anti-amyloid therapies [23].

How significantly will amyloid PET demand increase, once the therapy becomes available in the EU? According to very recent estimates of the Canada’s Drug Agency, 0.3% of the elderly population (above 65 years old) may be eligible for assessment for anti-amyloid therapies annually [24]. This rather *conservative* estimate assumes annual incidence of MCI and mild dementia of 6.4% (of the elderly population), with 1.2% presenting to health care and 0.5% undergoing assessment and diagnosis of early AD. Given a similar demographics in the EU and Canada, roughly 270.000 initial amyloid tests may be needed annually in the EU. In case of LP as preferred first test, 20 to 30% of the above population may require amyloid PET. If no preference is given, the demands will be even higher. In any scenario, the number of amyloid PET examinations will likely rise significantly as early as 2025.

How should the European nuclear medicine community prepare for this challenging task? First, we should re-assess capacities for amyloid PET. Specifically, national societies for nuclear medicine are encouraged to conduct a review of available and potential resources, in terms of scanning slots, personnel, and expertise. Second, we should closely interact with clinical partners at the national and European levels to better understand clinical needs and develop practical guidelines for patient selection and monitoring during anti-amyloid therapies. Third, in cooperation with clinical societies, we should find a consensus on several practical issues related to amyloid PET imaging. For example, what is the value of early perfusion scan as marker of neural injury and tau surrogate marker? What is the optimal image acquisition and analysis of repeated amyloid PET? What is the value of image quantification relative to visual reading? What is the value of FDG and tau PET in the context of anti-amyloid therapies?

In conclusion, the approval of Lecanemab represents an important milestone in AD treatment, necessitating a proactive response from the nuclear medicine community. Moving forward brings us to a future where, akin to current oncologic therapies, PET will be utilized for primary staging and re-staging of neurodegenerative proteinopathies.

## Data Availability

Not applicable.
